# Phenotypes of synovial fluid Treg cells in checkpoint blockade-related inflammatory arthritis

**DOI:** 10.3389/fonc.2025.1628790

**Published:** 2025-09-10

**Authors:** Heyu He, Ning Zhou, Kaidi Wu, Junhong Lin, Shaowei Zhou, Yuntao Gu, Xinjia Wang, Weidong Wang, Lichuan Mo, Chuanzhu Lv

**Affiliations:** ^1^ The Second Affiliated Hospital of Hainan Medical University, Haikou, Hainan, China; ^2^ The Emergency and Trauma College, Hainan Medical University, Haikou, Hainan, China; ^3^ Central People’s Hospital of Zhanjiang, Zhanjiang, Guangdong, China; ^4^ Department of Orthopedics and Spine Surgery, The Second Affiliated Hospital of Shantou University Medical College, Shantou, Guangdong, China; ^5^ Department of Orthopedics and Spine Surgery, Cancer Hospital of Shantou University Medical College, Shantou, Guangdong, China; ^6^ Department of Emergency Medicine Center, Sichuan Provincial People’s Hospital, University of Electronic Science and Technology of China, Chengdu, Sichuan, China; ^7^ Key Laboratory of Emergency and Trauma of Ministry of Education, Hainan Medical University, Haikou, Hainan, China

**Keywords:** immune-related adverse events, inflammatory arthritis, regulatory T cells, single-cell proteomics, cytokine secretion

## Abstract

**Objectives:**

Regulatory T (Treg) cells may become dysregulated in checkpoint blockade-related inflammatory arthritis (CBIA), and we aimed to profile phenotypes and cytokine-secreting patterns of Tregs in CBIA.

**Methods:**

Using a 77-protein panel, we here profiled and compared single-cell membrane proteomics of Treg cells in synovial fluid (SF) in 15 patients with active CBIA onset, 12 patients with active rheumatoid arthritis (RA), and 9 CBI-treated cancer patients with non-autoimmune inflammatory knee swelling. Microbead-sorted Treg subsets from CBIA patients underwent 32-cytokine panel secretome analysis. Peripheral blood (PB) Tregs from seven CBIA and six RA patients were similarly analyzed. Findings were correlated with the modified Clinical Disease Activity Index (mCDAI) in CBIA patients.

**Results:**

Unsupervised clustering revealed two atypical immune-activating Treg cell clusters common to both CBIA and RA patients, in which an immunoactivating (featuring ICOS^+^CD134^+^CD137^+^) cluster was distinct to CBIA patients. This immunoactivating cluster was found to have a positive correlation to the mCDAI in CBIA patients. In single-cell secreting proteomics of SF-derived Treg cells in CBIA patients, we found that clusters distinct to the immunoactivating cell group featured inflammatory cytokine secretion of mainly MCP1 and MCP4, which was validated by peripheral CBIA secreting proteomics (vs. RA, which preferentially secreted CCL11/CXCL10). Other non-immunoactivating cells mainly secreted immune-modulatory cytokines of IL-10, IL-4, and TGFB1. Consistently, the MCP1/MCP4^+^ polysecreting cluster proportion was also positively correlated with mCDAI.

**Conclusion:**

At the single-cell proteomic level, an atypical, MCP1/MCP4^+^ polysecreting immunoactivating Treg cell type is found to have a strong relation to clinical disease activity of CBIA.

## Introduction

In the past decade, checkpoint blockade immunotherapy (CBI) has become one of the cornerstones for anticancer therapy regimens and has succeeded in prolonging survival prognosis in many cancer types. On the other hand, CBI can variably cause adverse events, termed immune-related adverse events (irAEs), which significantly limit its clinical application for a number of sensitive patients (1). One of the most prevalent irAE types that are increasingly being studied currently is checkpoint blockade-related inflammatory arthritis (CBIA), which happens in 10% to 20% of patients receiving CBI-based therapy in oncology settings (2, 3). CBIA presents similar symptoms with most other rheumatic inflammatory arthritis conditions, including joint pain and swelling (3, 4). However, CBIA tends to have a persistent clinical course even with timely management and CBI cessation (5, 6). Steroid-based anti-inflammatory therapy may be efficient in acute settings, yet long-term steroids may raise concerns of compromised anticancer management (6, 7). Previous reports indicated that steroid management of second-line management is currently based upon clinical experience and expert opinions (5). It is thus necessary to further study pathogenesis for proper management.

One important mechanism of articular tissue injury brought by CBI is systemic off-target activation of T cells in addition to intratumoral checkpoint blockade, and intra-articular T-cell activation is suggested as one of the possible mechanisms of CBIA, although subtypes of T cells remain unclear so far (8). Regulatory T-cell lineages play key roles in maintaining immune homeostasis and self-tolerance in normal tissues, which become dysregulated in autoimmune diseases. As a subtype of CD4^+^ T cells, they express the key transcription factor FoxP3, which drives subsequent expression of immune-inhibitory markers and cytokines (9). Overall, typical Treg cell lineages are featured by the CD25^+^CD127^dim^ phenotype, secreting immune regulatory cytokines, including IL-4, IL-10, and TGFB (10). Research suggested autoimmune backgrounds of CBIA, and several pathological features may mimic rheumatoid arthritis (RA) at the cellular level (9). However, subsets of Treg lineages remain unidentified, which may reveal differences in dysregulated self-tolerance between the two pathology types and give further evidence on autoimmune backgrounds of CBIA (9). Previous single-cell studies of CBIA and RA suggested decreased proportions of Treg cells with atypical features, although subsets of Treg lineages have yet to be illustrated (9, 11).

In this study, we aim to identify the membranous protein expression differences of intra-articular Treg cells between CBIA and RA, as compared to non-autoimmune inflammatory controls (NICs), in order to find the CBIA-specific Treg cell phenotype. We designed an antibody-binding panel encompassing 77 surface and cellular markers of Treg cells, with reference to previous research cell phenotype studies (12–14). Next, by isolating key cell subtypes using laboratory techniques, we aim to identify the cytokine-secreting patterns at the single-cell level, exhibiting the functional status of the CBIA-specific Treg subset.

## Methods

### Settings and participants

In this prospective, single-center cohort study, we recruited participants diagnosed with new-onset CBIA after PD-1 or PD-L1 blockade treatment for cancers. Patients were enrolled from December 2020 to May 2023 at the Affiliated Cancer Hospital of Shantou University Medical College. Protocols of human research were reviewed and approved by the Institutional Review Boards of the Affiliated Cancer Hospital of Shantou University Medical College (No. 2023100; 14 July 2023), and the participants provided the required informed consent. Sampling procedures were performed according to the Helsinki Declaration. Information related to demographic variables, treatment history, and imaging studies of the participants was obtained from electronic medical records in which all participant data were de-identified. Reporting and analysis of data adhered to the Strengthening the Reporting of Observational Studies in Epidemiology (STROBE) checklist for cohort studies.

The purpose of this study was to explore distinct phenotypes of intra-articular Treg cells of CBIA patients during the active phase. Therefore, experiments were conducted using samples from the three groups of participants, namely, the CBIA, RA, and NIC groups, to achieve intergroup comparison. The diagnostic criteria of active CBIA are as follows: 1) active joint inflammation diagnosed by rheumatologists via medical checkups and serum inflammatory biomarkers of CRP as well as ESR, combined with imaging analysis of MRI or ultrasound; and 2) symptoms that occurred after checkpoint blocker initiation in cancer patients. The critical exclusion criteria included a history of or current status of autoimmune rheumatic diseases. Active RA was diagnosed according to the standards of the 2010 American College of Rheumatology/European League Against Rheumatism (ACR/EULAR). NIC patients were defined as CBI-treated cancer patients without CBIA or any other autoimmune conditions who underwent knee fluid aspiration due to traumatic or infectious knee swelling (non-autoimmune etiology).

### Patient sampling and assessment

All samples of CBIA and RA were collected during the active phase, with the time of disease onset recorded. Baseline information included sex, age, time to onset, cancer management, and arthritis management. As the Treg percentage modifies with age, all the study groups were age- and sex-matched. To analyze the relationship of key cell types with clinical relevance, we adopted the modified Clinical Disease Activity Index (mCDAI) to assess the levels of severity of CBIA during the onset phase. The mCDAI score was calculated by summing scores of the following: tender joint numbers, swollen joint numbers, the patient’s subjective assessment scores of 1 to 10, and the physician’s assessment scores of 1 to 10 (15). For both CBIA and RA, seropositive arthritis was defined as the presence of anti-citrullinated peptide antibodies (ACPA) as well as rheumatoid factor (RF). We additionally referred to the Common Terminology Criteria for Adverse Events (version 5.0) for symptom severity description and rating of CBIA.

Synovial fluid (SF) was sampled through knee arthrocentesis from patients with CBIA, RA, and NIC. Peripheral blood (PB) was drawn from the patients with CBIA and RA. SF and PB of CBIA and RA were obtained from the patients as discarded samples during clinical routine care in rheumatology departments. Samples of NIC patients were actively recruited from oncology settings as study volunteers with proper compensation. SF was collected during episodes of knee involvement unrelated to CBIA or autoimmune conditions. Cells were isolated from SF and PB via Ficoll-Paque gradient centrifugation. Treg^+^ cells were enriched by positive selection with CD4^+^CD25^+^CD127^dim^ microbeads (Miltenyi Biotec, Cologne, Germany, #130-094-775).

### Single-cell membrane proteomics and secreting proteomics

In the first sequencing step, single-cell membrane proteomics was carried out for Treg cells to identify distinct phenotypes by surface protein quantification using a total of 77 antibodies. Based on membrane proteomics results, SF-derived Tregs from CBIA patients underwent magnetic bead sorting: Treg subsets were sorted using CD137 microbeads to isolate immunoactivating cells. For comparative validation, PB-derived immunoactivating Tregs from CBIA and RA patients were similarly sorted using CD137 beads. In the second sequencing step, single-cell secreting proteomics was performed on SF- and PB-derived immunoactivating Tregs using the Single-Cell Secretome Adaptive Immune Chip - 4 (Human, 32-plex) panel. As such, the aim of this study was to profile cytokine and membrane phenotypes of distinct cell types at the single-cell proteomic level.

Sample preparation protocols were published previously (16, 17). Cell staining for membrane proteomics was performed using the Single Cell Multiplexing Kit (#633781, BD Biosciences, Franklin Lake, New Jersey, USA) and a master mix of 77 oligo-conjugated AbSeq antibodies (BD Biosciences, Franklin Lake, New Jersey, USA, see **Supplementary Table 1**) in PBS + 2% FBS for 45 min on ice. After three washes with cold BD sample buffer, cells were resuspended at 20,000–30,000 cells/mL and loaded onto BD Rhapsody cartridges (#633733; BD Biosciences, Franklin Lake, New Jersey, USA) for single-cell capture. An immunoactivating Treg subset was isolated using CD137 microbeads (Miltenyi Biotec, Cologne, Germany, #130-093-476) through a 15-min 4°C incubation in PBS/0.5% BSA/2 mM EDTA buffer (cell density ≤ 10^8^/mL; 10 μL beads per 10^7^ cells), followed by column-based separation where microbead^−^ cells were collected in flow-through, and microbead^+^ cells were eluted after column removal. Similarly, immunoactivating Treg subsets from PBMC were sorted using the same microbeads by positive selection. All sorted subsets were cryopreserved at >95% viability (Ficoll-Paque Plus; GE Healthcare, Chicago, Illinois, USA). Samples were thawed and then cultured in a complete RPMI medium (Fisher Scientific, Massachusetts, USA) at a density of 1 * 105 cells/mL, and viable cells were purified from dead cells with Ficoll-Paque Plus medium (GE Healthcare, Chicago, Illinois, USA). Cell suspension was loaded with 1,000–2,000 cells per chip onto a Single-Cell Secretome Adaptive Immune Chips (Bruker Cellular Analysis, ISOCODE-1001-4) with a 32-plex panel and incubated at 37°C, 5% CO_2_ for 24 h overnight (18, 19) (**Supplementary Table 2**). Secreted proteins were captured and quantified via fluorescence ELISA detection on the IsoLight platform with IsoSpeak software analysis (18).

### Single-cell data analysis

Data processing protocols have been previously published (18, 19). Identical synovial fluid volumes were processed uniformly across all patients. Treg frequencies were normalized by quantifying CD45^+^ viable cells per sample to ensure cross-sample comparability. Cleansing and auto-clustering were performed with the “Seurat” package on R (version 4.3.1) statistics. Non-barcoded cells and any antibody covering <20 cells were excluded from the analysis. Dimension reduction was carried out with principal component analysis (PCA) using all quality-controlled proteins. Then, Uniform Manifold Approximation and Projection (UMAP) was adopted for unsupervised auto-clustering. For membrane proteomics (CBIA, RA, and NIC), UMAP used 11 principal components (PCs) with a clustering resolution of 0.3. For secreting proteomics of immunoactivating Tregs in CBIA, UMAP used six PCs (resolution = 0.1). For secreting proteomics of immunoactivating Tregs between CBIA and RA, UMAP projections utilized five PCs (resolution = 0.1). The “FindAllMarkers” embedding was adopted for marker protein identification in each cluster, with a 0.25 difference from all other clusters, with a threshold for cell proportion set at 0.25. Global scaling was performed prior to log transformation and centering from counts of barcoded antibodies. Annotation of a single-cell membrane proteomics-defined cluster was performed by referencing CellMarker (version 2.0) (20).

### Statistics

Continuous variables were visualized with violin and dot plots, with statistical computation performed on R statistics (version 4.3.3) and GraphPad Prism (version 9.5.1, GraphPad Software). Comparable data were analyzed through a non-parametric Wilcoxon signed-rank test, with *p*-values adjusted for multiple comparisons using Benjamini–Hochberg (BH) correction. Pearson correlation analysis was carried out for cluster proportion and mCDAI scores for each patient.

## Results

### Baseline information and cell clustering

We first aimed to test cell markers of SF Treg cells in CBIA patients, distinct from two control groups of RA and NIC. For this, the single-cell membranous marker panels of CD4^+^CD25^+^CD127^dim^ cells were tested for 15 CBIA patients, 12 RA patients, and 11 NIC volunteers. The CBIA group included nine men and six women, with a median age of 54 (quartile range, 40–69) years, with a variety of cancer types and CBI types. These patients had a median onset time since the start of CBI of 5 (1–23) weeks, with a median mCDAI score of 21 (14–28). They were all treated with intravenous steroids combined with other anti-inflammatory therapies, with 10 patients having protracted clinical responses. All patients had negative serology of ACPA or RF. The demographic and treatment information for the CBIA, RA, and NIC groups is shown in **Table 1**.

**Table 1:** Demographic and treatment information for the CBIA, RA, and NIC groups.

Demographic and treatment information for the CBIA group													
Patient no.	Sex	Age	Cancer type	CBI type	Onset time^a^	Treatment	Remission	mCDAI	ACPA	RF	Serum ANA	CTCAE grade	CRP (mg/dL)
CBIA_1	M	62	NSCLC	Camrelizumab	5	LC+NSAIDs	Rapid	26	Neg.	Neg.	Neg.	3	110.23
CBIA_2	M	52	SCLC	Pembrolizumab	4	LC+NSAIDs	Protracted	16	Neg.	Neg.	Neg.	1	13.52
CBIA_3	M	49	NSCLC	Camrelizumab	11	LC+NSAIDs	Protracted	14	Neg.	Neg.	Neg.	1	14.13
CBIA_4	M	59	NSCLC	Nivolumab	17	LC+NSAIDs	Rapid	24	Neg.	Neg.	Neg.	3	84.21
CBIA_5	M	45	HCC	Toripalimab	3	LC+NSAIDs	Protracted	25	Neg.	Neg.	Pos.	3	94.23
CBIA_6	F	54	TNBC	Pembrolizumab	20	LC+NSAIDs	Protracted	18	Neg.	Neg.	Neg.	2	15.3
CBIA_7	F	40	EC	Sintilimab	9	LC+NSAIDs+MTX	Protracted	14	Neg.	Neg.	Neg.	2	26.96
CBIA_8	M	62	HCC	Sintilimab	8	LC+NSAIDs	Protracted	21	Neg.	Neg.	Neg.	2	15.53
CBIA_9	F	43	NSCLC	Camrelizumab	4	LC+NSAIDs	Rapid	28	Neg.	Neg.	Neg.	3	92.34
CBIA_10	M	54	HCC	Pembrolizumab	10	LC+MTX	Protracted	22	Neg.	Neg.	Neg.	2	21.53
CBIA_11	F	69	NPC	Sintilimab	23	LC+NSAIDs	Protracted	23	Neg.	Neg.	Neg.	2	40.11
CBIA_12	F	55	TNBC	Nivolumab	5	LC+MTX	Rapid	24	Neg.	Neg.	Neg.	3	45.23
CBIA_13	M	48	UC	Toripalimab	3	LC+NSAIDs	Protracted	15	Neg.	Neg.	Neg.	2	28.27
CBIA_14	M	42	EC	Pembrolizumab	1	LC+NSAIDs	Protracted	21	Neg.	Neg.	Pos.	2	24.14
CBIA_15	F	60	TNBC	Durvalumab	2	LC+NSAIDs	Rapid	20	Neg.	Neg.	Pos.	2	18.74
Demographic and treatment information in the RA group													
Patient no.	Sex	Age	Disease duration (years)	CRP (mg/dL)	Prior DEMARDs	Current DEMARDs	NSAIDs	mCDAI	ACPA	RF	Serum ANA		
RA_1	M	44	6	79.09	MTX	MTX	No	24	Neg.	Neg.	Neg.	–	–
RA_2	M	51	8	36	MTX	MTX	No	25	Neg.	Pos.	Neg.	–	–
RA_3	F	44	8	35	MTX	MTX	No	25	Pos.	Neg.	Neg.	–	–
RA_4	M	47	4	94.54	MTX	Combined	Yes	15	Pos.	Neg.	Neg.	–	–
RA_5	F	59	5	107.06	MTX	MTX	No	17	Pos.	Pos.	Neg.	–	–
RA_6	F	49	5	12.64	Biological	MTX	No	17	Neg.	Pos.	Neg.	–	–
RA_7	F	52	4	28.36	Biological	MTX	Yes	14	Neg.	Pos.	Neg.	–	–
RA_8	F	56	6	12.12	MTX	Biological	Yes	20	Neg.	Neg.	Neg.	–	–
RA_9	F	48	3	27.39	MTX	Biological	No	18	Pos.	Neg.	Neg.	–	–
RA_10	M	57	3	58.37	MTX	MTX	No	19	Pos.	Pos.	Neg.	–	–
RA_11	M	44	3	23.3	MTX	MTX	No	20	Neg.	Neg.	Neg.	–	–
RA_12	F	60	6	67.16	MTX	combined	No	22	Pos.	Pos.	Neg.	–	–
Demographic and treatment information in the NIC group													
Patient no.	Sex	Age	Cancer type	CBI type	–	–	–	–	–	–	–	–	–
NIC_1	M	57	UC	Toripalimab	–	–	–	–	–	–	–	–	–
NIC_2	F	60	TNBC	Camrelizumab	–	–	–	–	–	–	–	–	–
NIC_3	M	60	NPC	Camrelizumab	–	–	–	–	–	–	–	–	–
NIC_4	M	57	NSCLC	Sintilimab	–	–	–	–	–	–	–	–	–
NIC_5	M	46	NSCLC	Sintilimab	–	–	–	–	–	–	–	–	–
NIC_6	F	49	UC	Durvalumab	–	–	–	–	–	–	–	–	–
NIC_7	F	59	HCC	Pembrolizumab	–	–	–	–	–	–	–	–	–
NIC_8	M	44	GC	Pembrolizumab	–	–	–	–	–	–	–	–	–
NIC_9	M	45	NPC	Sintilimab	–	–	–	–	–	–	–	–	–
NIC_10	F	59	GC	Pembrolizumab	–	–	–	–	–	–	–	–	–
NIC_11	F	46	HCC	Camrelizumab	–	–	–	–	–	–	–	–	–

^a^Onset time since the start of CBI (weeks).

CBIA, checkpoint blockade-related inflammatory arthritis; RA, rheumatoid arthritis; NIC, non-autoimmune inflammatory control; M, male; F, female; NSCLC, non-small cell lung cancer; SCLC, small cell lung cancer; HCC, hepatocellular carcinoma; TNBC, triple-negative breast cancer; EC, endometrial cancer; NPC, nasopharyngeal carcinoma; UC, urogenital carcinoma; GC, gastric cancer; LC, local steroid; NSAIDs, non-steroidal anti-inflammatory drugs; MTX, methotrexate; mCDAI, modified Clinical Disease Activity Index; ACPA, anticitrullinated peptide antibodies; RF, rheumatoid factor; ANA, antinuclear antibody; CTCAE, common terminology criteria for adverse events; CRP, C-reactive protein; Neg., negative; Pos., positive; Biological, biological DEMARDs; combined, combined MTX, and biological DEMARDs

**Figure 1:**
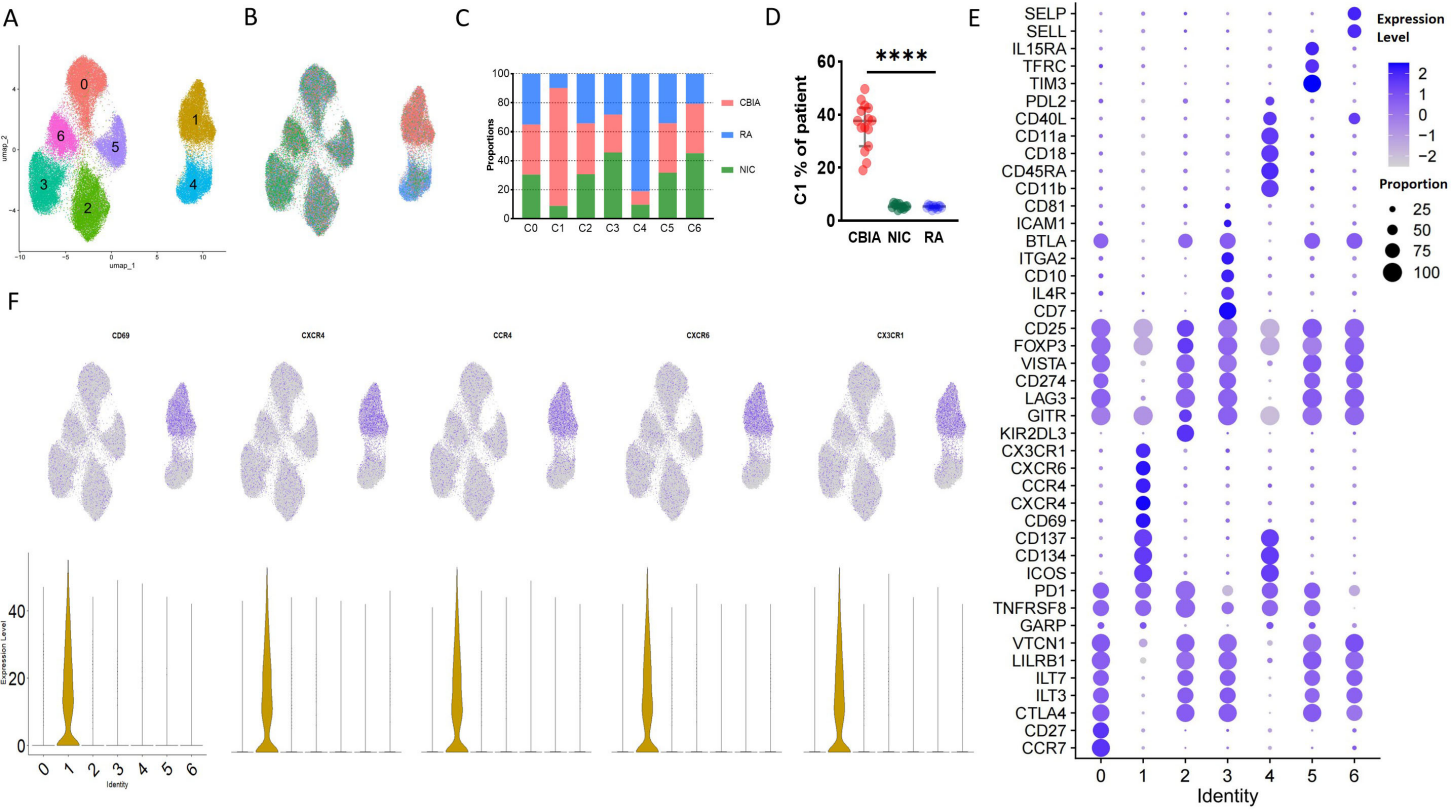
Single-cell membrane proteomics-defined clustering of Treg cells in all samples. **(A)** Membrane proteomics-defined UMAP plot visualizing Treg cell clusters, related to **Supplementary Figure 1A**. **(B)** Membrane proteomics-defined UMAP plot visualizing Treg cells from different groups, related to **Supplementary Figure 1B**. **(C)** Bar plot showing cell proportions in each proteomics-defined Treg cluster and group, related to **Supplementary Figure 1B**. **(D)** Patient-level variation of membrane proteomics-defined cluster 1 in each group, related to **Supplementary Figure 1C**. **(E)** Dot plot showing expression levels and cell proportions in each cluster, with purple dots exhibiting expression levels and size exhibiting proportions of expressing cells. **(F)** UMAP plot visualizing the expression of featured membrane markers in cluster 1. ****: P<0.0001.

Overall, the combined SF single-cell membranous proteomic data contained 60,508 quality-controlled cell lineages of all three groups, including 23,337 cells from the CBIA group, 19,973 cells from the RA group, and 17,198 cells from the NIC group. The number of cells isolated from individual patients is provided in **Supplementary Table 3**. Based on unsupervised clustering, seven clusters were identified and visualized as UMAP plots (**Figure 1A**, **Supplementary Figure 1A**), with the largest cluster containing 11,136 cells and the smallest cluster containing 5,934 cells (**Supplementary Figure 1B**). Cluster 1 was heavily populated with cells from CBIA patients, while cluster 4 was heavily populated with cells from the RA group (**Figures 1B, C**). Other clusters had no significant discrepancy among the three groups (**Figure 1C**). Patient-level analysis revealed ≥2-fold differences in clusters 1 and 4 relative to other clusters (**Figure 1D**, **Supplementary Figure 1C**). These findings preliminarily suggested different cell phenotypes of clusters 1 and 4 for the CBIA and RA groups, in contrast to the NIC group.

### Treg cells from the CBIA and RA groups exhibited common and distinct immunoactivating phenotype markers

Unsupervised clustering allowed us to further identify the specific markers associated with the cell clusters defined by differentially expressed protein (DEP) levels by cross-cluster comparison. With Seurat packages, we plotted a dot plot showing the 10 highest DEPs of each cluster (**Figure 1E**). Overall, all clusters expressed high levels of Treg feature markers, including CD3, CD4, CD25, and FOXP3, and low levels of IL-7R (**Figure 1E**, **Supplementary Figure 2A**). Interestingly, all clusters expressed medium levels of CD103, a marker indicating local antigen expression. According to the general classification principles of T-cell staging, we then annotated these clusters by maturation stage based upon CD45RA and CCR7: central memory Tregs (CD45RA^−^CCR7^+^, cluster 0), effector memory (CD45RA^−^CCR7^−^, clusters 1, 2, 3, 5, and 6), and terminally effector (CD45RA^+^CCR7^−^, cluster 4) (**Figure 1F**, **Supplementary Figure 2B**). CCR7 and CD45RA reflected the functional status and distribution of Treg cells, respectively, in which CCR7 was associated with tissue localization of lymphocytes while CD45RA was widely adopted for differentiating naive versus mature cells (21).

Clusters 1 and 4, which were two distinct clusters for CBIA and RA, exhibited significantly different phenotypes. Compared to other clusters, they both had significantly diminished levels of expression of classic inhibitory markers of Treg cells, including but not limited to CTLA4, VISTA, LAG3, and VTCN1 (**Figure 1E**, **Supplementary Figures 3A–C**). On the other hand, they both shared high levels of expression of stimulating markers, including CD137, CD134, and ICOS (**Figure 1E**, **Supplementary Figure 3D**), thus defined as an immunoactivating cell cluster. These findings preliminarily suggested that the two specific clusters of Treg cells in RA and CBIA may be atypical toward immune stimulation effects, compared to the typical immunoinhibitory phenotype. When separately analyzing their cluster features, we found that cluster 1 was characterized by several chemotaxis markers, including CX3CR1, CCR4, CXCR4, and CD69 (**Figures 1E, F**). Interestingly, cluster 4 featured CD18 and CD11a expression (**Figure 1E**, **Supplementary Figure 4**), which are two important components of the heterodimer LFA1. This evidence suggested that although both clusters were stimulatory atypical Treg cells, CBIA-specific Tregs were characterized by chemotactic phenotypes while RA-specific Tregs featured adhesion phenotypes (**Supplementary Figure 5**).

### Distinct secreting cytokines of immunoactivating+ Tregs in CBIA patients relative to RA patients

**Figure 2:**
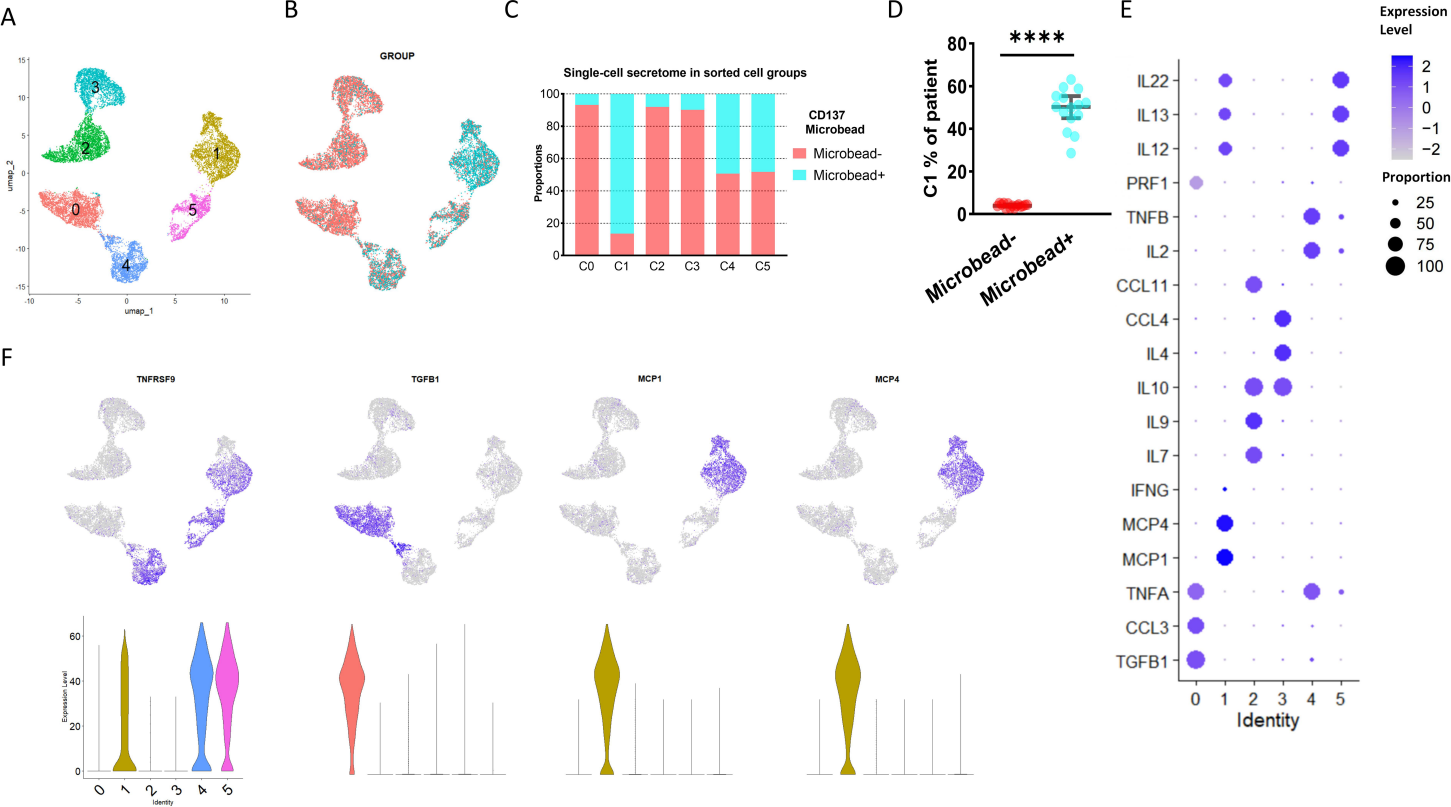
Single-cell secreting proteomics-defined clusters of Treg cell samples in CBIA patients at onset. **(A)** Single-cell secreting proteomics-defined UMAP plot visualizing cell clusters from both microbead^+^ and microbead^−^ isolated cells combined, related to **Supplementary Figure 6A**. **(B)** Single-cell secreting proteomics-defined UMAP plot visualizing cells categorized by microbead^+^ and microbead^−^ groups, related to **Supplementary Figure 6B**. **(C)** Bar plot showing cell proportions of each cluster in the microbead^+^ and microbead^−^ cell samples, related to **Supplementary Figure 6B**. **(D)** Patient-level variation of secreting proteomics-defined cluster 1 in the microbead^+^ and microbead^−^ cell samples, related to **Supplementary Figure 6C**. **(E)** Dot plot showing single-cell secreting levels and cytokine-secreting cell proportions in each cluster, with purple dots exhibiting secreting levels and size exhibiting proportions of secreting cells. **(F)** UMAP plot visualizing expression of featured cytokines, related to **Supplementary Figure 6C**. CBIA, blockade-related inflammatory arthritis. ****: P<0.0001.

Considering the immunophenotypes of cluster 1 (annotated as CBIA-specific immune-activating Tregs, distinct from the CBIA group) in our study that exhibited an atypical phenotype of Treg cells, and that this activating type may be associated with CBIA pathology status, we then sought to investigate the secreting cytokines of such cells in CBIA patients to determine their secreting functions at the cellular level. In CBIA patient samples, we found that ICOS^+^CD134^+^CD137^+^ was almost exclusively expressed in this cluster, and as such, CD137 microbeads were adopted to positively and negatively sort cells, yielding immunoactivating and CD137^−^ cell groups. These sorted cells underwent single-cell secreting proteomics in a commercial 32-protein chip (**Supplementary Table 2**). These cytokines encompass a variety of functions, including cytotoxic effects, chemotaxis, cell stimulation, immune modulation, and inflammation. We finally procured quality-controlled 15,819 cells for the single-cell secreting proteomic protocol, including 5,284 immunoactivating and 10,535 CD137^−^ cells. The number of cells isolated from individual patients is provided in **Supplementary Table 3**. By means of unsupervised clustering, these cells auto-clustered into six clusters, as seen in the UMAP plot (**Figure 2A**, **Supplementary Figure 6A**), with the largest cluster containing 3,126 cells and the smallest cluster containing 1,554 cells.

Clusters 0, 2, and 3 were heavily populated with secreting cells of the CD137^−^ group, while cluster 1 was heavily populated with cells from the immunoactivating group (**Figures 2B, C**). Cluster 1 exhibited at least a two-fold difference at the patient level (**Figure 2D**, **Supplementary Figure 6C**). Other clusters had no significant discrepancy. These findings preliminarily suggested different secreting functions of immunoactivating cells as opposed to CD137^−^ cells. Then, these clusters were annotated based on cytokine secretion (**Figure 2E**). Specifically, cluster 1 was featured by MCP1 and MCP4, which are key cytokines for inflammation (**Figure 2F**). Consistent with typical Treg cell functions, however, clusters 0, 2, and 3 were featured for high levels of IL4, IL-10, and TGFB1 secretion, exhibiting immune modulation functions in CD137^−^ cells (**Figures 2E, F**, **Supplementary Figure 6D**). Overall, these findings validated that SF-derived immunoactivating cells in CBIA patients, rather than CD137^−^ cells, played key roles in inflammation at the cellular function level.

Single-cell membrane proteomic analysis revealed that both CBIA and RA exhibited atypical stimulation effects rather than the classical regulatory effects of Treg. To further identify the differences in the immunoactivating Treg cells in CBIA and RA, we obtained peripheral blood from seven CBIA patients and six RA patients from the currently participating patients via the Treg and CD137 magnetic bead isolation kit. These sorted cells were subjected to single-cell secreting proteomics of 32 proteins (**Supplementary Table 2**). We finally procured 11,229 cells for single-cell secreting proteomics, including 5,748 cells in the CBIA group and 5,481 cells in the RA group (see **Supplementary Table 3** for specific cell numbers per person). By means of unsupervised clustering, these cells auto-clustered into four clusters, as seen in the UMAP plot (**Supplementary Figure 7A**), with the largest cluster containing 3,412 cells and the smallest cluster containing 2,285 cells (**Supplementary Figure 7B**).

Cluster 0 was heavily populated with secreting cells from immunoactivating Treg cells of CBIA, while cluster 1 was heavily populated with cells from the immunoactivating Treg group of RA (**Supplementary Figures 7B–D**). Patient-level analysis revealed ≥4-fold differences in clusters 0 and 1 relative to other clusters (**Supplementary Figures 7E, F**). Other clusters had no significant discrepancy. These findings preliminarily suggested different secreting functions of immunoactivating Tregs in CBIA as opposed to RA. Then, these clusters were annotated based on cytokine secretion (**Supplementary Figure 7G**). Specifically, cluster 0 was featured by MCP1 and MCP4, which are key cytokines for inflammation. However, cluster 1 was featured for high levels of CCL11 and CXCL10 secretion, exhibiting chemoattractive functions (**Supplementary Figure 7G**). These indicate that immunoactivating Treg cells exhibit distinct secretory cytokines in patients with CBIA from those in RA patients, which may represent different immune mechanisms at the cellular function level.

### Synovial fluid-derived immune-activating Treg cell is associated with disease activity

**Figure 3:**
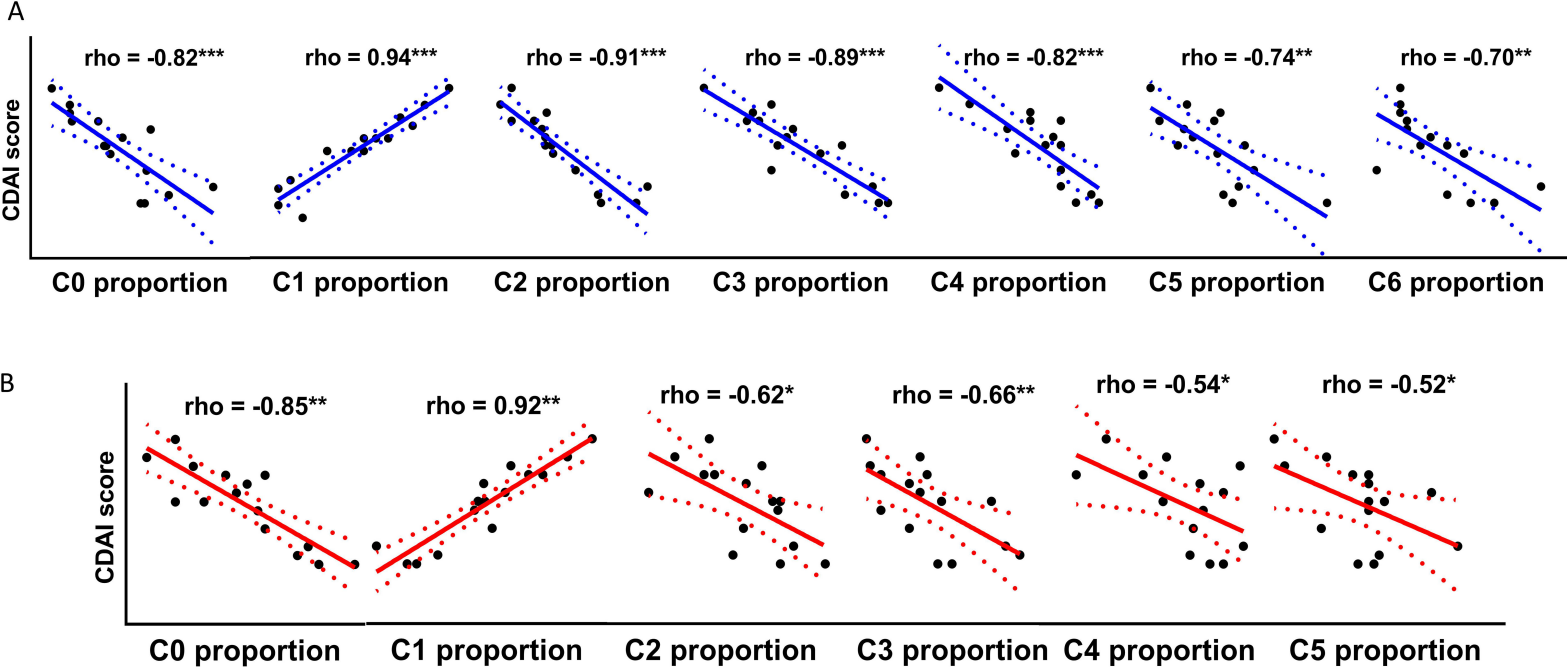
Correlative plots of cluster proportions with mCDAI scores in CBIA patients. **(A)** Correlative plots of single-cell membrane proteomics-defined cluster proportions and mCDAI scores, related to **Supplementary Figure 8**. **(B)** Correlative plots of single-cell secretome proteomics-defined cluster proportions of immunoactivating Tregs and mCDAI scores, related to **Supplementary Figure 9**. CBIA, blockade-related inflammatory arthritis; mCDAI, modified Clinical Disease Activity Index.

We illustrated the immunophenotypes of Treg cells distinct in CBIA patients, and these distinct cells were immunoactivating cells with active secreting functions. To what extent these microenvironments were associated with clinical features has not been resolved so far. Here, we made an association analysis of our findings with disease activity, mainly designated with the mCDAI score, to illustrate the clinical implications of single-cell clustering. In single-cell membrane proteomics-defined clusters, cluster 1 proportions were positively associated with mCDAI scores (**Figure 3A**) and also with clinical CRP values and CTCAE ratings, which represent the severity of adverse effects (**Supplementary Figure 8**). In SF-derived immunoactivating Tregs from CBIA patients, cluster 1 proportions were positively associated with mCDAI scores (**Figure 3B**) and also with clinical CRP values and CTCAE ratings (**Supplementary Figure 9**). In PBMC-derived immunoactivating Tregs from CBIA and RA, cluster 0 and 1 proportions were positively associated with mCDAI scores, respectively, with different cellular functions (**Supplementary Figures 7H, I**). All the other cluster proportions were negatively associated with mCDAI scores. These findings coherently validated that immune-activated Treg cells were positively correlated with disease activity.

## Discussion

In this study, through single-cell membranous and secreting proteomic experiments, we profiled the phenotypes and secreting cytokines of intra-articular Treg cells of CBIA patients in comparison with those cells from the RA and NIC patient groups. We identified predominantly an MCP1/MCP4^+^ polysecreting immunoactivating Treg cell subset, exhibiting both phenotypical and functional immune activation patterns, and this subset showed a positive association with clinical disease activity of CBIA patients with mCDAI scores. This finding substantiated the prior reported hypothesis of Treg cell dysregulation in CBIA etiology, which is recognized as an autoimmune T-cell-mediated iatrogenic condition (4, 22). Considering the atypical phenotypes and corresponding secreting functions found in CBIA (23), our study illustrated the landscape of Treg cells in inflammatory arthritis and shed light on the subset functions of Treg cells in a sizable cohort.

Treg cells play key roles in modulating immune stability and inflammatory inhibition in normal tissues, with classic expression of many types of surface markers suggesting immune inhibition, including VISTA, CTLA4, ILT3, and ILT7 (24). However, Treg cells may become dysregulated in autoimmune conditions, such as RA, and a literature review suggested that RA patients exhibited decreased Treg cell populations with decreased expression of surface immune inhibitory markers (24–26). Consistently, we identified that Treg cells specific to RA patients expressed decreased levels of many inhibitory or immune-modulating markers. On the other hand, previous studies on CBIA exhibited decreased Treg populations in synovial fluid, but the exact phenotypes have been poorly understood (27). In this study, we showed that intra-articular Treg cells in CBIA showed both similar and distinct immune-activating signatures with Tregs from RA patients. For instance, Treg cells in CBIA shared with the RA groups with features of increased expression of activation markers, including CD137 (TNFRSF9 or 4-1BB), CD134, and ICOS, while both groups shared features of decreased expression of immune-inhibitory markers. As special findings in our research, distinct features of Treg cells in CBIA patients not shared with RA included high levels of chemotaxis markers, such as CXCR4, CXCR6, and CCR4, while RA patients exhibited increased levels of CD11a, CD11b, CD18, and importantly, CD45RA. These findings together suggested different Treg cell activities from RA patients (28), and future research is encouraged to resolve the difference.

One key finding of the Treg cell in our study is the notable corresponding secreting cytokine analysis of the atypical Treg cells after identifying that the distinct phenotype was specific to intra-articular Treg cells in CBIA patients. By comparing the single-cell secreting proteomics in immunoactivating Treg cells with that of negatively sorted cells, we found that immunoactivating cell groups were functionally consistent with their phenotypes, with multiple inflammatory cytokine secretion, including mainly MCP1 and MCP4. Conversely, cytokines with immune-inhibitory functions were found in immunoactivating cell groups, including cell clusters that secreted IL-10, IL-4, and TGFB1. Critically, our integrated PB-derived secretome proteomics demonstrates that immunoactivating Tregs in CBIA preferentially secrete pro-inflammatory MCP1/MCP4, whereas RA immunoactivating Tregs produce the chemokines CCL11/CXCL10—functionally aligning with synovial fluid observations and underscoring disease-specific Treg polarization. These results suggested that phenotypes and secreting functions of intra-articular Treg cells are consistent at the protein level and that CBIA-specific Treg cells exhibited mainly immunostimulating and pro-inflammatory functions. Finally, in CBIA patients, we correlated their clinical disease activity with proportions of single-cell-defined clusters and illustrated positive associations, underscoring the role of MCP1/MCP4 secreting immunoactivating Tregs in CBIA disease progression.

The biological significance of MCP1/MCP4 secretion by CBIA-specific Tregs warrants emphasis. In RA patients, MCP1 (CCL2) and MCP4 (CCL13) are established mediators of synovitis: MCP1 recruits monocytes via CCR2, driving macrophage infiltration and osteoclastogenesis that accelerates joint destruction, while MCP4/CCL13 directly activates fibroblast-like synoviocyte proliferation through extracellular signal-regulated kinase mitogen-activated protein kinase cascade signaling, amplifying cartilage degradation (29, 30). However, CBIA reflects acute T-cell dysregulation triggered by immune checkpoint inhibitors (ICIs) (31). Critically, our study reveals a divergent pathogenic role in CBIA: here, immunoactivating Tregs specifically overproduce MCP1/MCP4 (rather than RA-associated chemokines like CCL11/CXCL10), creating a feedforward loop that sustains synovial inflammation. This aligns with emerging evidence on irAE pathogenesis. In neurotoxicity induced by ICIs, elevated MCP1 primes T-cell hyperactivation and disrupts immune tolerance—a process mirrored in our CBIA cohort, where MCP1/MCP4^+^ Tregs correlate with CTCAE grades (31). Thus, dysregulated Tregs in CBIA may actively fuel inflammation via MCP1/MCP4, a phenomenon unreported in conventional autoimmune arthritis. However, this finding is limited by sample size and should be read with caution, and a larger-scale cohort is encouraged to empower our findings.

Therapeutically, targeting the MCP1/MCP4–CCR2/CCR3 axis holds promise for CBIA. CCR2 inhibitors suppress monocyte migration in RA models (32), but their efficacy in CBIA may extend to blocking Treg–T-cell crosstalk. Notably, combined blockade of MCP1 and MCP4 failed to synergistically inhibit monocyte recruitment in RA synovium, possibly because CCL13 and CCL2 jointly use the chemokine receptor CCR2 on monocytes, and thus, CCL13 blockade was as effective as CCL2 (29). Therefore, a similar strategy could disrupt the inflammatory circuit of CBIA.

### Limitation

Although we illustrated the immune landscape of intra-articular Treg cells and identified disease-specific cell clusters, several limitations are present. First, as mentioned, although we obtained a large dataset enough for phenotype profiling at a single-cell level, the patient sample size is relatively low for clinical correlation. Secondly, the cell counts of each patient were relatively low because of cell loss from sampling difficulties and single-cell protocols. Thirdly, techniques using membrane and secreting proteomics were limited by the number of antibodies manufactured commercially. Unlike transcriptomic sequencing spanning a wide range of RNAs, this technique caused biased profiling by antibody numbers, and as such, cell cluster identification may be inaccurate. A further limitation is the absence of arthrosis or osteoarthritis controls, which would have provided a stronger comparative context for distinguishing inflammation-specific Treg dysregulation in the CBIA and RA cohorts. Lastly, the absence of orthogonal validation (e.g., flow cytometry for surface markers, immunohistochemistry for tissue localization) and independent longitudinal cohorts necessitates cautious interpretation.

### Conclusion

We profiled the phenotypes and cytokines of synovial fluid-derived Treg cells of CBIA patients and made comparisons with the RA and NIC patient groups. We identified an MCP1/MCP4^+^ polysecreting immunoactivating Treg subset as a distinct pathogenic contributor in CBIA, showing significant associations with clinical disease activity and systemic inflammation markers.

## Data Availability

The datasets presented in this study can be found in online repositories. The names of the repository/repositories and accession number(s) can be found below: https://www.scidb.cn/en/s/InIbee.

## References

[1] Suijkerbuijk KPM van Eijs MJM van Wijk F Eggermont AMM. Clinical and translational attributes of immune-related adverse events. Nat Cancer. (2024) 5:557–71. doi: 10.1038/s43018-024-00730-3, PMID: 38360861

[2] McCarter KR Wolfgang T Arabelovic S Wang X Yoshida K Banasiak EP. Mortality and immune-related adverse events after immune checkpoint inhibitor initiation for cancer among patients with pre-existing rheumatoid arthritis: a retrospective, comparative, cohort study. Lancet Rheumatol. (2023) 5:e274–e83. doi: 10.1016/S2665-9913(23)00064-4, PMID: 37841635 PMC10571093

[3] Cunningham-Bussel A Wang J Prisco LC Martin LW Vanni KMM Zaccardelli A. Predictors of rheumatic immune-related adverse events and *de novo* inflammatory arthritis after immune checkpoint inhibitor treatment for cancer. Arthritis Rheumatol. (2022) 74:527–40. doi: 10.1002/art.41949, PMID: 34397169 PMC8847547

[4] Bass AR Xie F Jannat-Khah D Ghosh N Chan KK Saxena A. Incidence of checkpoint inhibitor-associated inflammatory arthritis, immunomodulation and mortality in cancer patients on immunotherapy: a retrospective cohort study. Rheumatol (Oxford England). (2024) 64(4):1637–42. doi: 10.1093/rheumatology/keae343, PMID: 38889288 PMC11962947

[5] Braaten TJ Brahmer JR Forde PM Le D Lipson EJ Naidoo J. Immune checkpoint inhibitor-induced inflammatory arthritis persists after immunotherapy cessation. Ann rheumatic diseases. (2020) 79:332–8. doi: 10.1136/annrheumdis-2019-216109, PMID: 31540935 PMC7031780

[6] Bass AR Abdel-Wahab N Reid PD Sparks JA Calabrese C Jannat-Khah DP. Comparative safety and effectiveness of TNF inhibitors, IL6 inhibitors and methotrexate for the treatment of immune checkpoint inhibitor-associated arthritis. Ann rheumatic diseases. (2023) 82:920–6. doi: 10.1136/ard-2023-223885, PMID: 37019614 PMC10330686

[7] Petit PF Daoudlarian D Latifyan S Bouchaab H Mederos N Doms J. Tocilizumab provides dual benefits in treating immune checkpoint inhibitor-associated arthritis and preventing relapse during ICI rechallenge: the TAPIR study. Ann oncology: Off J Eur Soc Med Oncol. (2024) 36(1):43–53. doi: 10.1016/j.annonc.2024.08.2340, PMID: 39241964

[8] Kim ST Chu Y Misoi M Suarez-Almazor ME Tayar JH Lu H. Distinct molecular and immune hallmarks of inflammatory arthritis induced by immune checkpoint inhibitors for cancer therapy. Nat Commun. (2022) 13:1970. doi: 10.1038/s41467-022-29539-3, PMID: 35413951 PMC9005525

[9] Zhou Z Zhou X Jiang X Yang B Lu X Fei Y. Single-cell profiling identifies IL1Bhi macrophages associated with inflammation in PD-1 inhibitor-induced inflammatory arthritis. Nat Commun. (2024) 15:2107. doi: 10.1038/s41467-024-46195-x, PMID: 38453911 PMC10920757

[10] Knochelmann HM Dwyer CJ Bailey SR Amaya SM Elston DM Mazza-McCrann JM. When worlds collide: Th17 and Treg cells in cancer and autoimmunity. Cell Mol Immunol. (2018) 15:458–69. doi: 10.1038/s41423-018-0004-4, PMID: 29563615 PMC6068176

[11] Wang R Singaraju A Marks KE Shakib L Dunlap G Adejoorin I. Clonally expanded CD38hi cytotoxic CD8 T cells define the T cell infiltrate in checkpoint inhibitor–associated arthritis. Sci Immunol. (2023) 8:eadd1591. doi: 10.1126/sciimmunol.add1591, PMID: 37506196 PMC10557056

[12] Povoleri GAM Durham LE Gray EH Lalnunhlimi S Kannambath S Pitcher MJ. Psoriatic and rheumatoid arthritis joints differ in the composition of CD8+ tissue-resident memory T cell subsets. Cell Rep. (2023) 42:112514. doi: 10.1016/j.celrep.2023.112514, PMID: 37195862 PMC10790246

[13] Tay C Tanaka A Sakaguchi S. Tumor-infiltrating regulatory T cells as targets of cancer immunotherapy. Cancer Cell. (2023) 41:450–65. doi: 10.1016/j.ccell.2023.02.014, PMID: 36917950

[14] Haroun-Izquierdo A Vincenti M Netskar H van Ooijen H Zhang B Bendzick L. Adaptive single-KIR(+)NKG2C(+) NK cells expanded from select superdonors show potent missing-self reactivity and efficiently control HLA-mismatched acute myeloid leukemia. J immunotherapy Cancer. (2022) 10(11):e005577. doi: 10.1136/jitc-2022-005577, PMID: 36319065 PMC9628692

[15] Aletaha D Smolen J. The Simplified Disease Activity Index (SDAI) and the Clinical Disease Activity Index (CDAI): a review of their usefulness and validity in rheumatoid arthritis. Clin Exp Rheumatol. (2005) 23:S100–8. doi: 10.1016/j.berh.2007.02.004, PMID: 16273793

[16] Erickson JR Mair F Bugos G Martin J Prlic M. AbSeq protocol using the nano-well cartridge-based rhapsody platform to generate protein and transcript expression data on the single-cell level. STAR Protoc. (2020) 1(2):100062. doi: 10.1016/j.xpro.2020.100092, PMID: 33000001 PMC7523635

[17] Shahi P Kim SC Haliburton JR Gartner ZJ Abate AR. Abseq: Ultrahigh-throughput single cell protein profiling with droplet microfluidic barcoding. Sci Rep. (2017) 7:44447. doi: 10.1038/srep44447, PMID: 28290550 PMC5349531

[18] Liu D Paczkowski P Mackay S Ng C Zhou J. Single-cell multiplexed proteomics on the isoLight resolves cellular functional heterogeneity to reveal clinical responses of cancer patients to immunotherapies. Methods Mol Biol. (2020) 2055:413–31. doi: 10.1007/978-1-4939-9773-2_19, PMID: 31502163

[19] Abbas H Alaniz Z Mackay S Cyr M Zhou J Issa G. Single-cell polyfunctional proteomics of CD4 cells from patients with AML predicts responses to anti-PD-1-based therapy. Blood advances. (2021) 5:4569–74. doi: 10.1182/bloodadvances, PMID: 34555853 PMC8759127

[20] Hu C Li T Xu L Zhang X Li F Bai J. CellMarker 2.0: an updated database of manually curated cell markers in human/mouse and web tools based on scRNA-seq data. Nucleic Acids Res. (2022) 51(D1):D870–D876. doi: 10.1093/nar/gkac947, PMID: 36300619 PMC9825416

[21] Miyara M Yoshioka Y Kitoh A Shima T Wing K Niwa A. Functional delineation and differentiation dynamics of human CD4+ T cells expressing the FoxP3 transcription factor. Immunity. (2009) 30:899–911. doi: 10.1016/j.immuni.2009.03.019, PMID: 19464196

[22] Chan KK Bass AR. Monitoring and management of the patient with immune checkpoint inhibitor-induced inflammatory arthritis: current perspectives. J Inflammation Res. (2022) 15:3105–18. doi: 10.2147/JIR.S282600, PMID: 35642215 PMC9148583

[23] Chen M Li H Qu B Huang X. The roles of T cells in immune checkpoint inhibitor-induced arthritis. Aging disease. (2024) 16(4):2100–19. doi: 10.14336/AD.2024.0546, PMID: 39122457 PMC12221389

[24] Talib M Gyebrovszki B Bőgér D Csomor R Mészáros A Fodor A. Helper T cells are hyperactive and contribute to the dysregulation of antibody production in patients with rheumatoid arthritis. Int J Mol Sci [Internet]. (2024) 25. doi: 10.3390/ijms251810190, PMID: 39337675 PMC11431999

[25] Li B Su R Guo Q Su R Gao C Li X. Differential immunological profiles in seronegative versus seropositive rheumatoid arthritis: Th17/Treg dysregulation and IL-4. Front Immunol. (2024) 15:1447213. doi: 10.3389/fimmu.2024.1447213, PMID: 39290695 PMC11405332

[26] Nishiyama T Ohyama A Miki H Asashima H Kondo Y Tsuboi H. Mechanisms of age-related Treg dysfunction in an arthritic environment. Clin Immunol (Orlando Fla). (2024) 266:110337., PMID: 39111562 10.1016/j.clim.2024.110337

[27] Rajak P. Immune checkpoint inhibitors: From friend to foe. Toxicol Rep. (2025) 14:102033. doi: 10.1016/j.toxrep.2025.102033, PMID: 40353246 PMC12063143

[28] Vetsika EK Fragoulis GE Kyriakidi M Verrou KM Tektonidou MG Alissafi T. Insufficient PD-1 expression during active autoimmune responses: a deep single-cell proteomics analysis in inflammatory arthritis. Front Immunol. (2024) 15:1403680. doi: 10.3389/fimmu.2024.1403680, PMID: 38911848 PMC11190177

[29] Hintzen C Quaiser S Pap T Heinrich PC Hermanns HM. Induction of CCL13 expression in synovial fibroblasts highlights a significant role of oncostatin M in rheumatoid arthritis. Arthritis Rheumatism. (2009) 60:1932–43. doi: 10.1002/art.24602, PMID: 19565514

[30] Chen L Wu X Zhong J Li D. L161982 alleviates collagen-induced arthritis in mice by increasing Treg cells and down-regulating Interleukin-17 and monocyte-chemoattractant protein-1 levels. BMC Musculoskelet Disord. (2017) 18:462. doi: 10.1186/s12891-017-1819-3, PMID: 29145862 PMC5691865

[31] Möhn N Mahjoub S Duzzi L Narten E Grote-Levi L Körner G. Monocyte chemoattractant protein 1 as a potential biomarker for immune checkpoint inhibitor-associated neurotoxicity. Cancer Med. (2023) 12:9373–83. doi: 10.1002/cam4.5695, PMID: 36794673 PMC10166892

[32] Moadab F Khorramdelazad H Abbasifard M. Role of CCL2/CCR2 axis in the immunopathogenesis of rheumatoid arthritis: Latest evidence and therapeutic approaches. Life Sci. (2021) 269:119034. doi: 10.1016/j.lfs.2021.119034, PMID: 33453247

