# Supplementary material for: Phenotypes of synovial fluid Treg cells in checkpoint blockade-related inflammatory arthritis
Source: Front Oncol. 2025 Sep 10;15:1628790. doi: 10.3389/fonc.2025.1628790 (PMC12457161; doi:10.3389/fonc.2025.1628790)
Supplement: Supplementary file 1 [file DataSheet1.docx]

Supplementary Materials

Supplementary Table 1. Antibody lists in single-cell membrane proteomics.

| **Number** | **M**arker^*^ | **L**og | **D**escription |
| --- | --- | --- | --- |
| 1 | CX3CR1 | 940216 | Hu CX3CR1 Olgo AHS0125 2A9-1 25Tst |
| 2 | IL7R | 940012 | Hu CD127 Ab-O AHS0028 HIL-7R-M21 25Tst |
| 3 | CD36 | 940224 | Hu CD36 Olgo AHS0135 CLB-IVC7 25Tst |
| 4 | CD96 | 940272 | Hu CD96 Olgo AHS0194 6F9 25Tst |
| 5 | CXCR6 | 940234 | Hu CXCR6 Olgo AHS0148 13B 1E5 25Tst |
| 6 | CD69 | 940019 | Hu CD69 Ab-O AHS0010 FN50 25Tst |
| 7 | CXCR4 | 940056 | Hu CD184 Ab-O AHS0060 12G5 25Tst |
| 8 | CCR4 | 940047 | Hu CD194 Ab-O AHS0038 1G1 25Tst |
| 9 | CTLA4 | 940034 | Hu CD152 Ab-O AHS0017 BNI3 25Tst |
| 10 | CD47 | 940082 | Hu CD47 Ab-O AHS0087 B6H12 25Tst |
| 11 | ITGA1 | 940094 | Hu CD49a Ab-O AHS0101 SR84 25Tst |
| 12 | ITGA2 | 940087 | Hu CD49b Ab-O AHS0093 12F1 25Tst |
| 13 | ITGA4 | 940059 | Hu CD49d Ab-O AHS0063 9F10 25Tst |
| 14 | CD4 | 940001 | Hu CD4 Ab-O AHS0032 SK3 25Tst |
| 15 | CD10 | 940045 | Hu CD10 Ab-O AHS0051 HI10A 25Tst |
| 16 | IL4R | 940092 | Hu CD124 Ab-O AHS0098 HIL4R-M57 25Tst |
| 17 | ICAM1 | 940072 | Hu CD54 Ab-O AHS0076 HA58 25Tst |
| 18 | PSG1 | 940088 | Hu CD66 Ab-O AHS0094 B1.1/CD66 25Tst |
| 19 | CD81 | 940052 | Hu CD81 Ab-O AHS0021 JS-81 25Tst |
| 20 | CD9 | 940078 | Hu CD9 Ab-O AHS0082 M-L13 25Tst |
| 21 | THY1 | 940032 | Hu CD90 Ab-O AHS0045 5E10 25Tst |
| 22 | CD18 | 940086 | Hu CD18 Ab-O AHS0091 6.7 25Tst |
| 23 | CD11a | 940077 | Hu CD11a Ab-O AHS0081 HI111 25Tst |
| 24 | CD11b | 940008 | Hu CD11b Ab-O AHS0005 M1/70 25Tst |
| 25 | CD45RA | 940011 | Hu CD45RA Ab-O AHS0009 HI100 25Tst |
| 26 | TFRC | 940275 | Hu CD71 Olgo AHS0197 L01.1 25Tst |
| 27 | IL15RA | 940290 | Hu CD215 Olgo AHS0212 JM7A4 25Tst |
| 28 | CD44 | 940039 | Hu CD44 Ab-O AHS0054 G44-26 25Tst |
| 29 | NOTCH1 | 940292 | Hu NOTCH1 Olgo AHS0214 MHN1-519 25Tst |
| 30 | NOTCH3 | 940298 | Hu NOTCH3 Olgo AHS0221 MHN3-21 25Tst |
| 31 | IL6R | 940090 | Hu CD126 Ab-O AHS0096 M5 25Tst |
| 32 | CSF2RB | 940385 | Hu CD131 Olgo AHS0263 3D7 25Tst |
| 33 | MET | 940222 | Hu C-MET Olgo AHS0132 3D6 25Tst |
| 34 | CSF2RA | 940311 | Hu CD116 Olgo AHS0238 hGMCSFR-M1 25Tst |
| 35 | SPN | 940278 | HU CD43 Olgo AHS0200 1G10 25Tst |
| 36 | CD58 | 940371 | Hu CD58 Olgo AHS0237 1C3 25Tst |
| 37 | PVR | 940102 | Hu CD155 Ab-O AHS0111 TX24 25Tst |
| 38 | ITGA5 | 940106 | Hu CD49E Ab-O AHS0118 IIA1 25Tst |
| 39 | CD63 | 940243 | Hu CD63 Olgo AHS0157 H5C6 25Tst |
| 40 | CD99 | 940214 | Hu CD99 Olgo AHS0123 TU12 25Tst |
| 41 | SELE | 940377 | Hu CD62E Olgo AHS0254 68-5H11 25Tst |
| 42 | PDPLN | 940270 | HU PDPLN Olgo AHS0192 LPMAB-17 25Tst |
| 43 | TNFRSF13B | 940285 | Hu CD267 Olgo AHS0207 1A1-K21-M22 25Tst |
| 44 | TNFRSF13C | 940284 | Hu CD268 Olgo AHS0206 11C1 25Tst |
| 45 | IL2RB | 940232 | Hu CD122 Olgo AHS0146 MIK-BETA3 25Tst |
| 46 | IL12RB1 | 940267 | Hu CD212 Olgo AHS0185 2.4E6 25Tst |
| 47 | CD3 | 940000 | Hu CD3 Ab-O AHS0033 SK7 25Tst |
| 48 | PD1 | 940015 | Hu CD279 Ab-O AHS0014 EH12.1 25Tst |
| 49 | GARP | 940217 | Hu GARP Olgo AHS0126 7B11 25Tst |
| 50 | TNFRSF8 | 940103 | Hu CD30 Ab-O AHS0114 BERH8 25Tst |
| 51 | ICAM2 | 940241 | Hu CD102 Olgo AHS0155 CBR-IC2/2 25Tst |
| 52 | EPHB2 | 940259 | Hu EPHB2 Olgo AHS0176 2H9 25Tst |
| 53 | CD5 | 940038 | Hu CD5 Ab-O AHS0047 UCHT2 25Tst |
| 54 | PDL2 | 940071 | Hu CD273 (PDL2) Ab-O AHS0075 MIH18 25Tst |
| 55 | ILT7 | 940252 | HU CD85G/ILT7 Olgo AHS0168 17G10.2 25Tst |
| 56 | CD274 | 940035 | Hu CD274 Ab-O AHS0004 MIH1 25Tst |
| 57 | ILT3 | 940261 | Hu CD85K (ILT3) Olgo AHS0179 ZM3.8 25Tst |
| 58 | BTLA | 940105 | Hu CD272 Ab-O AHS0052 J168-540 25Tst |
| 59 | CD40L | 940053 | Hu CD154 Ab-O AHS0077 TRAP1 25Tst |
| 60 | CD137 | 940055 | Hu CD137 Ab-O AHS0003 4B4-1 25Tst |
| 61 | CD134 | 940060 | Hu CD134 Ab-O AHS0013 ACT35 25Tst |
| 62 | ICOS | 940043 | Hu CD278 Ab-O AHS0012 DX29 25Tst |
| 63 | CD7 | 940029 | Hu CD7 Ab-O AHS0043 M-T701 25Tst |
| 64 | SELL | 940041 | Hu CD62L Ab-O AHS0049 DREG-56 25Tst |
| 65 | TIM3 | 940066 | Hu TIM-3 Ab-O AHS0016 7D3 25Tst |
| 66 | SELP | 940309 | Hu CD62P Olgo AHS0234 AC1.2 25Tst |
| 67 | LILRB1 | 940249 | Hu CD85J Olgo AHS0163 GHI/75 25Tst |
| 68 | VTCN1 | 940100 | Hu B7-H4 Ab-O AHS0108 MIH43 25Tst |
| 69 | VISTA | 940497 | Hu VISTA Olgo AHS0187 MIH65.RMAB 25Tst |
| 70 | KIR2DL3 | 940287 | Hu KIR-NKAT2 Olgo AHS0209 DX27 25Tst |
| 71 | CD25 | 940009 | Hu CD25 Ab-O AHS0026 2A3 25Tst |
| 72 | GITR | 940096 | Hu GITR Ab-O AHS0104 V27-580 25Tst |
| 73 | FOXP3 | 460039 | MS FoxP3 Olgo ACU7001 MF23 25Tst |
| 74 | CD103 | 940067 | Hu CD103 Ab-O AHS0001 BER-ACT8 25Tst |
| 75 | LAG3 | 940080 | Hu LAG-3 Ab-O AHS0018 T47-530 25Tst |
| 76 | CCR7 | 940394 | Hu CCR7(CD197) Olgo AHS0273 2-L1-A 25Tst |
| 77 | CD27 | 940018 | Hu CD27 Ab-O AHS0025 M-T271 25Tst |
| ^*^Five key functional reasons for the selection of 77-makers:  1) Treg Identity & Lineage Definition: CD3, CD4, FOXP3, CD25, CD45RA, CD27, CD103, GARP (to definitively identify Treg subsets).  2) Immune Checkpoints & Costimulation: CTLA4, PD1, LAG3, TIM3, BTLA, CD274 (PD-L1), PDL2, ICOS, CD137 (4-1BB), CD134 (OX40), GITR, CD40L, TNFRSF8 (CD30), TNFRSF13B (BAFFR), TNFRSF13C (BAFF), ILT3, ILT7, VTCN1, VISTA, LILRB1, KIR2DL3, PVR (CD155, TIGIT ligand) (to profile dysregulated inhibitory/stimulatory pathways).  3) Migration & Tissue Homing: CX3CR1, CXCR6, CXCR4, CCR4, CCR7, SELL (L-selectin), SELP (P-selectin ligand), SELE, CD44, ITGA1 (VLA-1), ITGA2 (VLA-2), ITGA4 (VLA-4), ITGA5 (VLA-5), CD11a/CD18 (LFA-1), CD11b (Mac-1), ICAM1, ICAM2, CD58 (LFA-3), CD99 (to elucidate Treg recruitment to inflamed joints).  4) Activation, Metabolism & Signaling: CD69, CD137 (TNFRSF9), CD71 (TFRC), CD36, MET, NOTCH1, NOTCH3, EPHB2, IL7R, IL4R, IL6R, IL15RA, IL2RB, IL12RB1, CSF2RA, CSF2RB, CD5, CD7, SPN, CD63 (to quantify Treg functional states).  5) Novel Functional Modulators: CD96, CD47, CD10, PSG1, CD81, CD9, THY1 (CD90), PDPLN (to explore unconventional mechanisms). | | | |

Supplementary Table 2. Antibody lists in single-cell secreting proteomics.

| **Number** | **Isoplexis panel^*^** | **Function** |
| --- | --- | --- |
| 1 | GZMB | Effect |
| 2 | IFNG | Effect |
| 3 | CCL3 | Effect |
| 4 | PRF1 | Effect |
| 5 | TNFA | Effect |
| 6 | TNFB | Effect |
| 7 | GMCSF | Stimulatory |
| 8 | IL2 | Stimulatory |
| 9 | IL5 | Stimulatory |
| 10 | IL7 | Stimulatory |
| 11 | IL8 | Stimulatory |
| 12 | IL9 | Stimulatory |
| 13 | IL12 | Stimulatory |
| 14 | IL15 | Stimulatory |
| 15 | IL21 | Stimulatory |
| 16 | CCL11 | Chemotaxis |
| 17 | CXCL10 | Chemotaxis |
| 18 | CCL4 | Chemotaxis |
| 19 | CCL5 | Chemotaxis |
| 20 | IL4 | Modulatory |
| 21 | IL10 | Modulatory |
| 22 | IL13 | Modulatory |
| 23 | IL22 | Modulatory |
| 24 | TGFB1 | Modulatory |
| 25 | TNFRSF9 | Modulatory |
| 26 | CD40L | Modulatory |
| 27 | IL1B | Inflammatory |
| 28 | IL6 | Inflammatory |
| 29 | IL17A | Inflammatory |
| 30 | IL17F | Inflammatory |
| 31 | MCP1 | Inflammatory |
| 32 | MCP4 | Inflammatory |
| ^*^Single-cell secreting proteomics was tested using Single-Cell Secretome Adaptive Immune Chips - 4 (Human) (Bruker Cellular Analysis, ISOCODE-1001-4). | | |

Supplementary Table 3. The number of cells obtained from each patient in each single-cell proteomics

| **Patients** | **SF membrane proteomics** | **SF secreting proteomics by CD137 bead** | | **PBMC secreting proteomics** |
| --- | --- | --- | --- | --- |
|  |  | **bead-** | **bead+** |  |
| CBIA_1 | 1838 | 679 | 457 | -- |
| CBIA_2 | 1162 | 682 | 227 | 801 |
| CBIA_3 | 1233 | 741 | 283 | -- |
| CBIA_4 | 1738 | 734 | 412 | 831 |
| CBIA_5 | 1669 | 712 | 367 | 768 |
| CBIA_6 | 1445 | 727 | 348 | 820 |
| CBIA_7 | 1390 | 711 | 275 | -- |
| CBIA_8 | 1531 | 753 | 339 | -- |
| CBIA_9 | 1822 | 665 | 447 | -- |
| CBIA_10 | 1578 | 646 | 391 | 800 |
| CBIA_11 | 1645 | 671 | 345 | 844 |
| CBIA_12 | 1665 | 711 | 403 | 884 |
| CBIA_13 | 1418 | 693 | 289 | -- |
| CBIA_14 | 1643 | 724 | 360 | -- |
| CBIA_15 | 1560 | 686 | 341 | -- |
| RA_1 | 1673 | -- | -- | -- |
| RA_2 | 1682 | -- | -- | 882 |
| RA_3 | 1577 | -- | -- | -- |
| RA_4 | 1600 | -- | -- | 876 |
| RA_5 | 1678 | -- | -- | 918 |
| RA_6 | 1653 | -- | -- | 975 |
| RA_7 | 1707 | -- | -- | 889 |
| RA_8 | 1571 | -- | -- | -- |
| RA_9 | 1757 | -- | -- | 941 |
| RA_10 | 1659 | -- | -- | -- |
| RA_11 | 1742 | -- | -- | -- |
| RA_12 | 1674 | -- | -- | -- |
| NIC_1 | 1612 | -- | -- | -- |
| NIC_2 | 1544 | -- | -- | -- |
| NIC_3 | 1554 | -- | -- | -- |
| NIC_4 | 1578 | -- | -- | -- |
| NIC_5 | 1528 | -- | -- | -- |
| NIC_6 | 1601 | -- | -- | -- |
| NIC_7 | 1623 | -- | -- | -- |
| NIC_8 | 1556 | -- | -- | -- |
| NIC_9 | 1532 | -- | -- | -- |
| NIC_10 | 1542 | -- | -- | -- |
| NIC_11 | 1528 | -- | -- | -- |


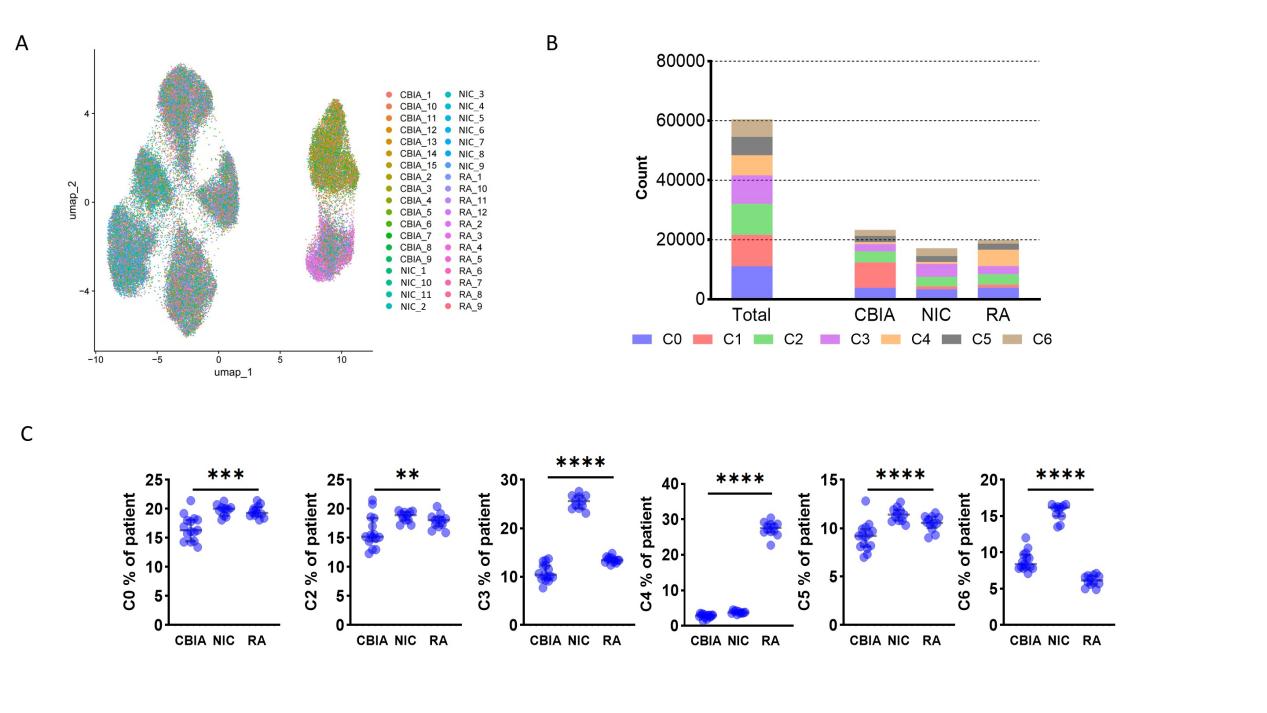


**Supplementary Figure 1. UMAP visualizations and patient-level variation in the single-cell membrane secretome proteomics**

A. UMAP plot visualizing single-cell membrane proteomics in cell samples of each patient in all 3 groups, related to Figure 1A.

B. Absolute cell count in each cluster categorized by all and each group, related to Figure 1B-C.

C. Patient-level variation in each cluster, related to Figure 1C-D.

**
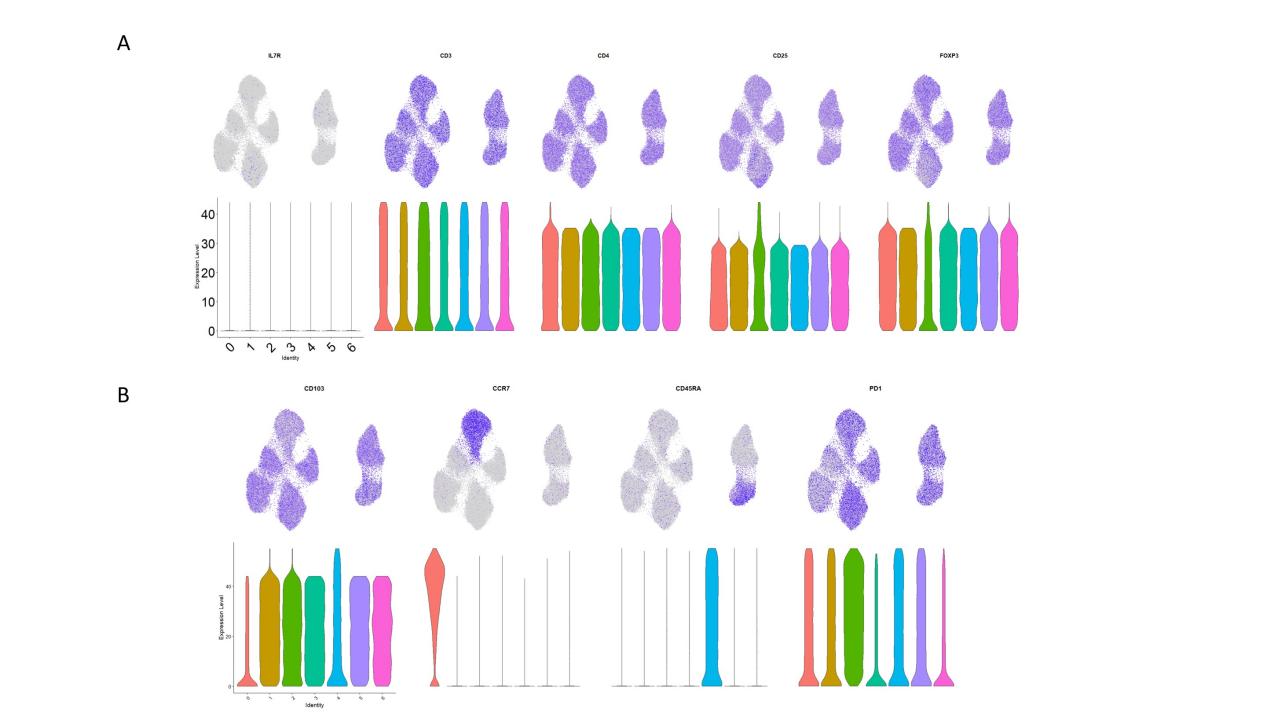
**

**Supplementary Figure 2. Protein expression and UMAP visualizations in markers used to define common features.**

A. UMAP plot visualizing expression of featured membrane markers used to define Treg (CD3+CD4+CD25+FOXP3+IL7R^dim^).

B. UMAP plot visualizing expression of featured membrane markers used to categorize T cell maturation states.


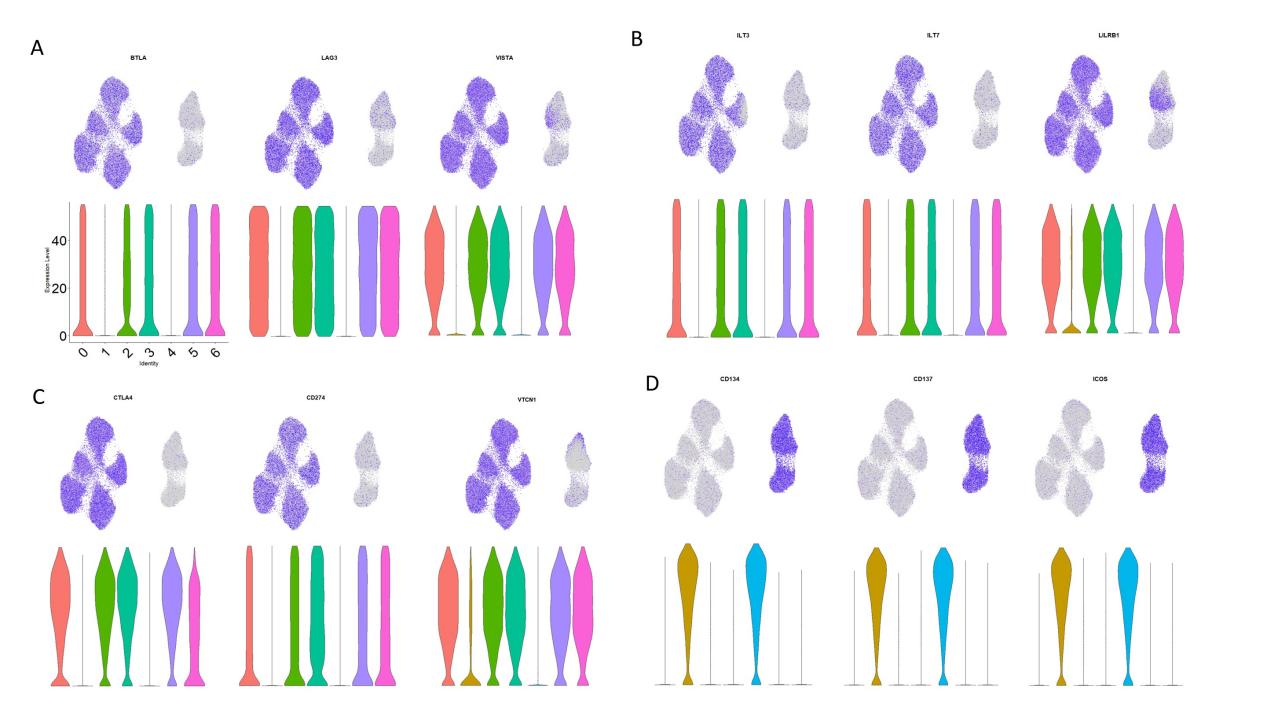


**Supplementary Figure 3. Protein expression and UMAP visualizations in markers common to cluster 1 and 4.**

A-C. UMAP plot visualizing low-expressed markers common to cluster 1 and 4.

D. UMAP plot visualizing high-expressed markers common to cluster 1 and 4.


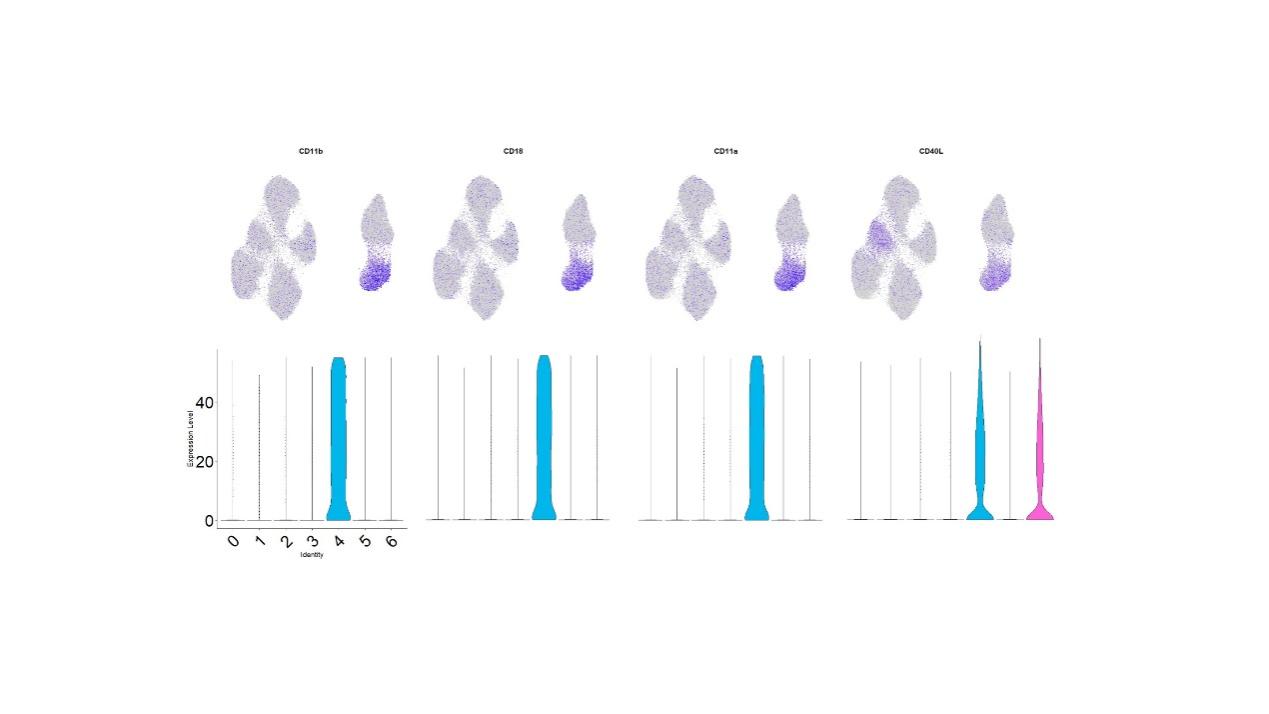


**Supplementary Figure 4. Protein expression and UMAP visualizations in markers distinct to cluster 4.**

**
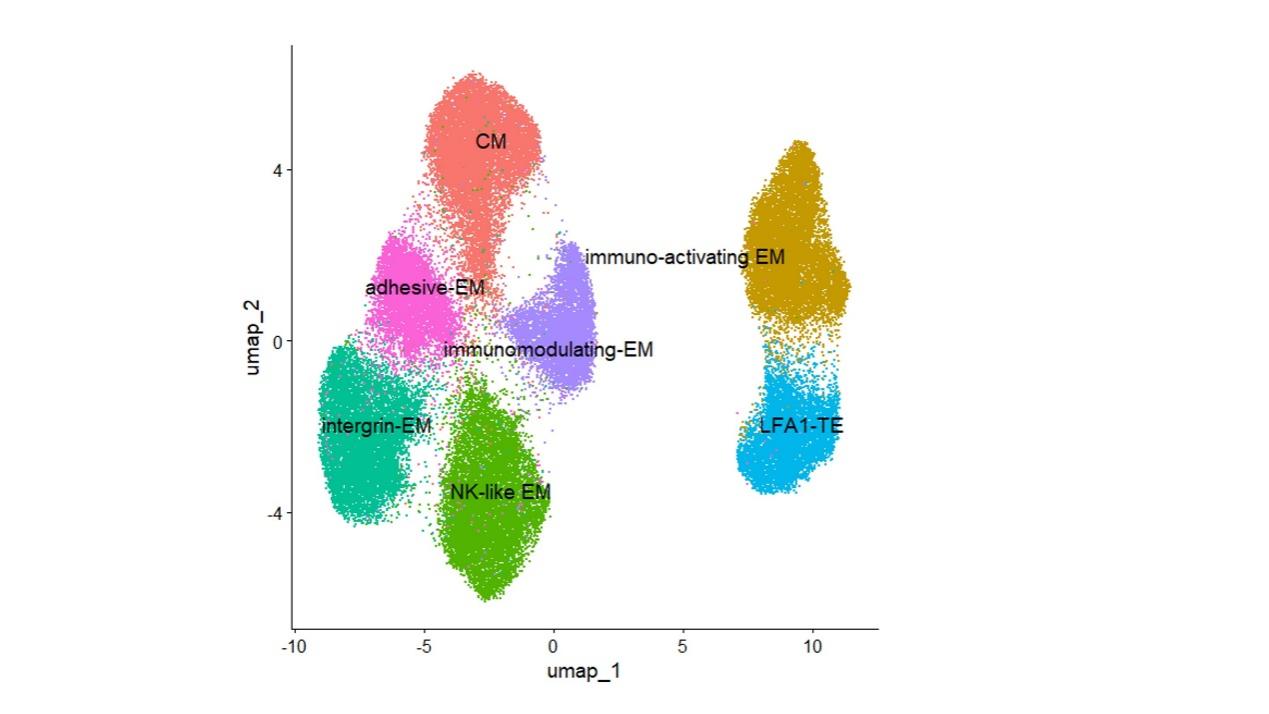
**

**Supplementary Figure 5. Treg cell cluster phenotype annotation visualized with UMAP plot.**

Abbr.: EM, Effector memory; CM, central memory; NK, natural killer; TM, terminal effector.


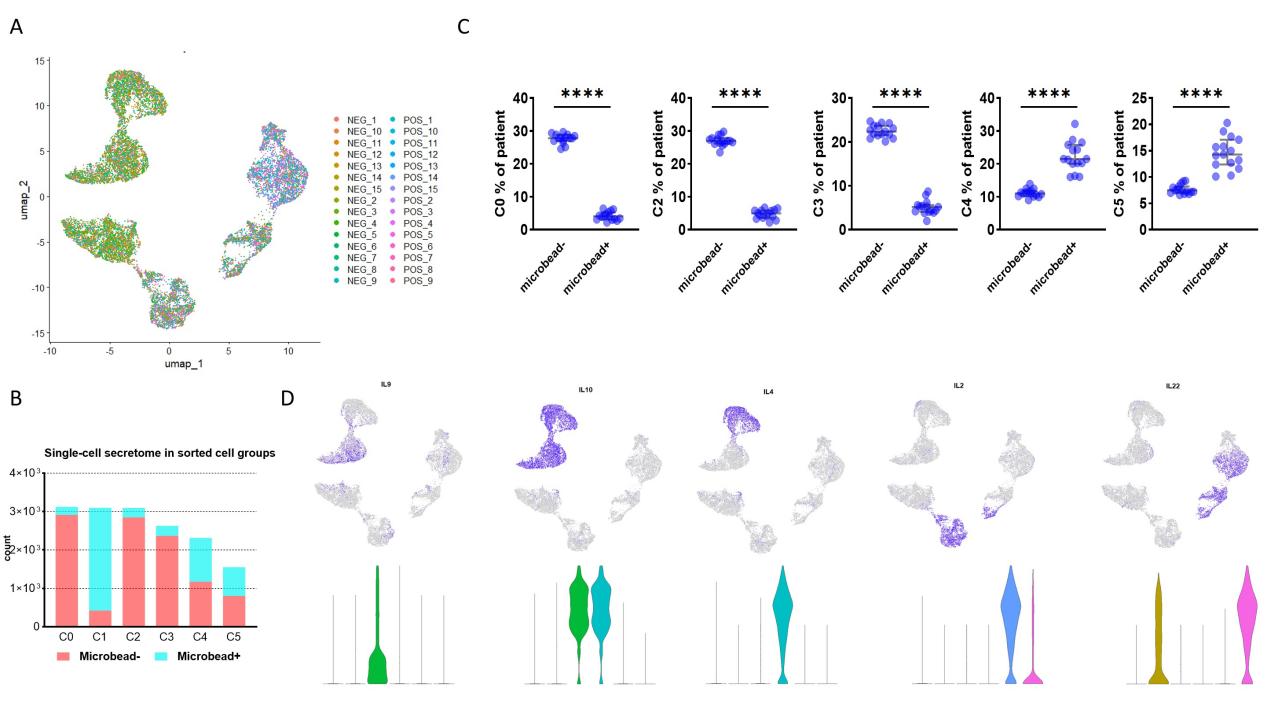


**Supplementary Figure 6. UMAP visualizations and patient-level variations in SF-derived single-cell secreting proteomics.**

A. UMAP plot visualizing single-cell secreting proteomics in cell samples of each patient in all groups, related to Figure 2A.

B. Absolute cell count in each group categorized by each cluster, related to Figure 2B-C.

C. Patient-level variation in each cluster, related to Figure 2C-D.

D. UMAP plot visualizing expression of featured cytokines in each cluster, related to Figure 2E-F.

**
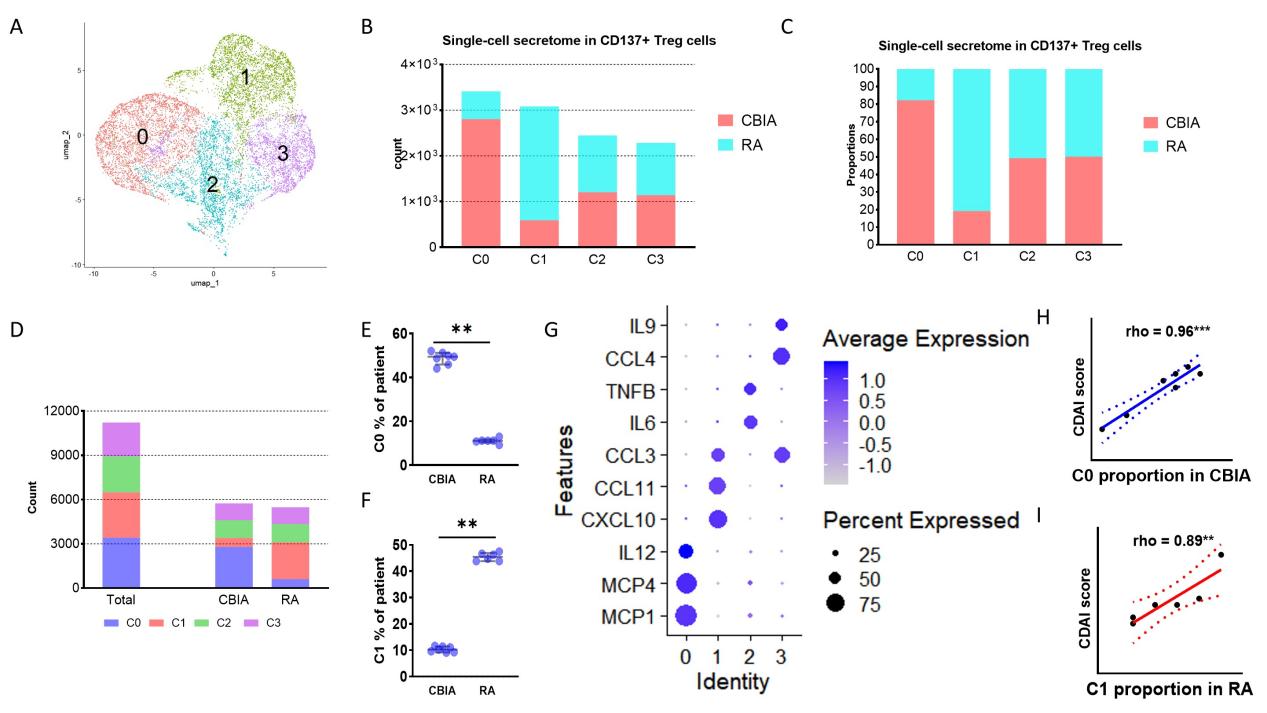
**

**Supplementary Figure 7. Protein expression and UMAP visualizations in single-cell secreting proteomics in PBMC-derived immuno-activating Tregs between CBIA and RA**

A. Single-cell secreting proteomics-defined UMAP plot visualizing cell clusters from both CBIA and RA immuno-activating cells combined.

B. Absolute cell count in each group categorized by each cluster.

C. Bar plot showing cell proportions of each cluster in the CBIA and RA immuno-activating cell samples.

D. Absolute cell count in each cluster categorized by all and each group, related to Figure 1B-C.

E-F. Patient-level variation in cluster 0 and cluster 1, which were two distinct clusters for CBIA and RA immuno-activating Tregs.

G. Dot plot showing expression levels and cell proportions in each cluster, with purple dot exhibiting expression levels and size exhibiting proportions of expressing cells.

H. Correlative plots of cluster 0 proportions in CBIA patients and mCDAI scores.

I. Correlative plots of cluster 1 proportions in RA patients and mCDAI scores.


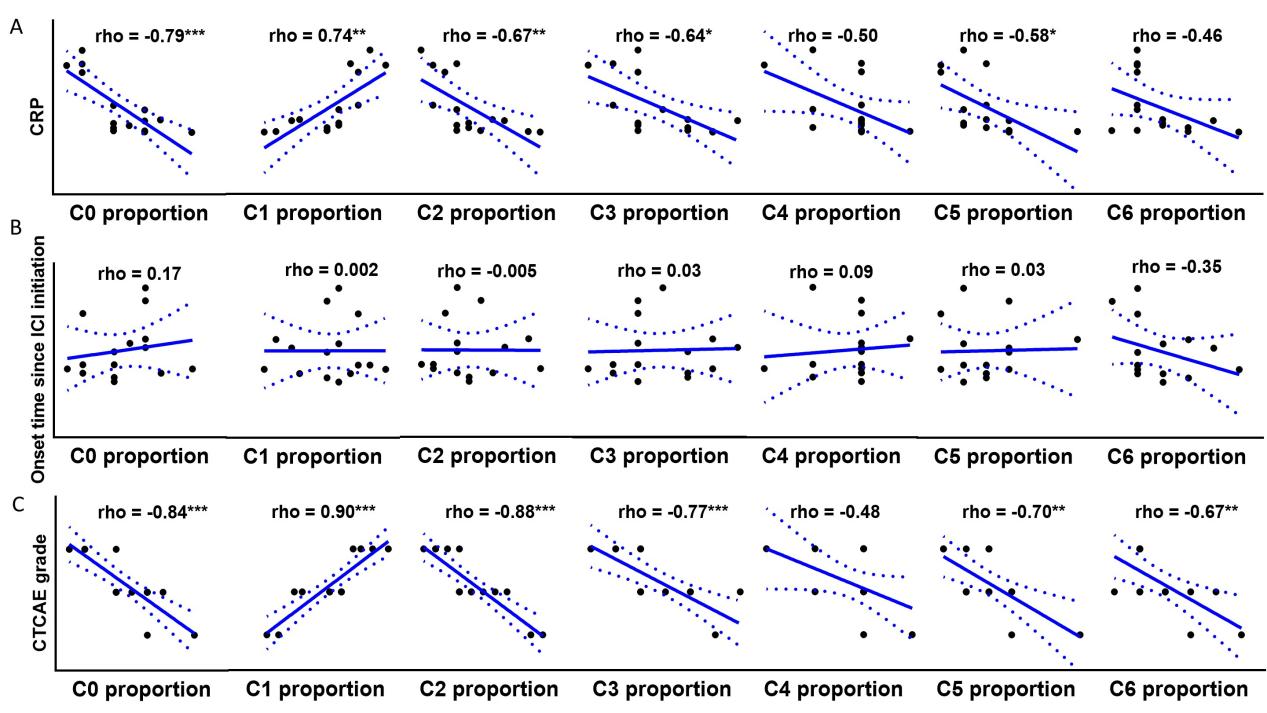


**Supplementary Figure 8. Correlative plots of cluster proportions of single-cell membrane proteomics with other clinical indicators in CBIA patients**

A. Correlative plots of cluster proportions and CRP value.

B. Correlative plots of cluster proportions and CBIA onset time since ICI initiation.

C. Correlative plots of cluster proportions and CTCAT grade.

**
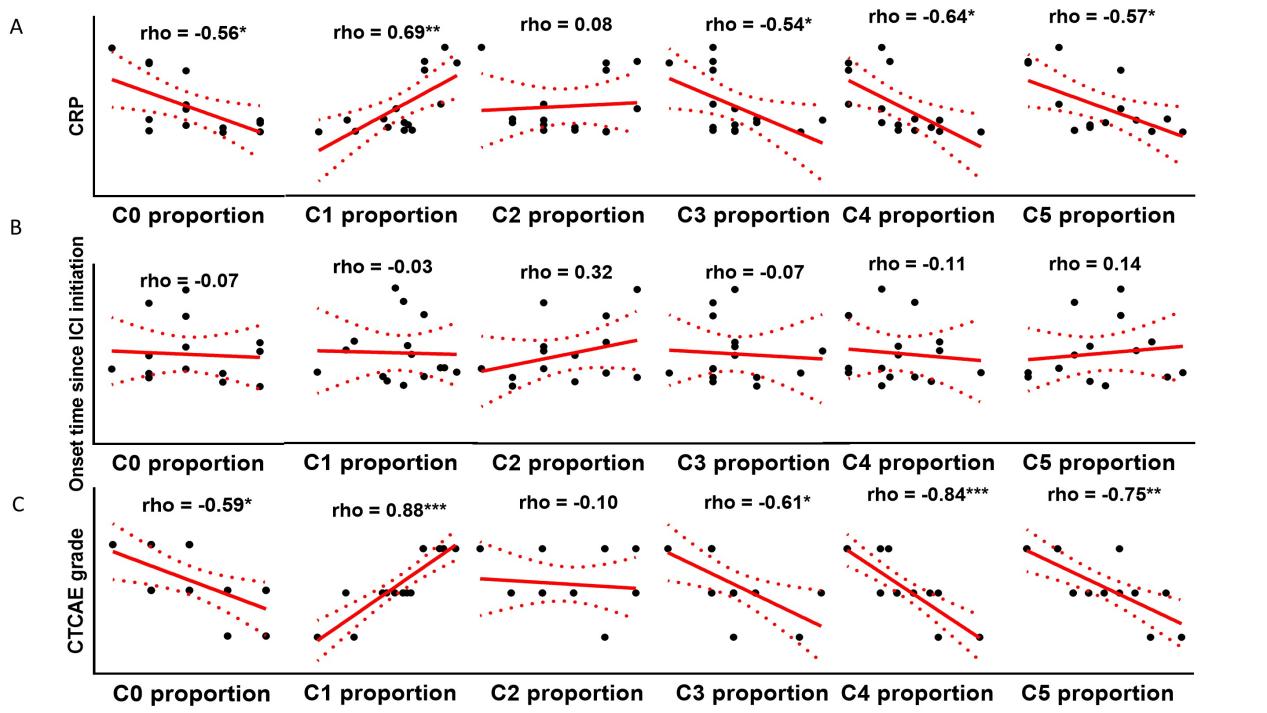
**

**Supplementary Figure 9. Correlative plots of single-cell secretome proteomics-defined cluster proportions of immuno-activating Tregs with other clinical indicators in CBIA patients**

A. Correlative plots of cluster proportions and CRP value.

B. Correlative plots of cluster proportions and CBIA onset time since ICI initiation.

C. Correlative plots of cluster proportions and CTCAT grade.
